# Effects of intracorneal ring segments implementation technique and design on corneal biomechanics and keratometry in a personalized computational analysis

**DOI:** 10.1038/s41598-021-93821-5

**Published:** 2021-07-13

**Authors:** Niksa Mohammadi Bagheri, Mahmoud Kadkhodaei, Shiva Pirhadi, Peiman Mosaddegh

**Affiliations:** 1 Department of Mechanical Engineering, Isfahan University of Technology, Isfahan, 84156-83111 Iran; 2 Department of Biomedical Engineering, Science and Research Branch, Islamic Azad University, Tehran, 14778-93855 Iran

**Keywords:** Biomedical engineering, Computational science, Engineering

## Abstract

The implementation of intracorneal ring segments (ICRS) is one of the successfully applied refractive operations for the treatment of keratoconus (kc) progression. The different selection of ICRS types along with the surgical implementation techniques can significantly affect surgical outcomes. Thus, this study aimed to investigate the influence of ICRS implementation techniques and design on the postoperative biomechanical state and keratometry results. The clinical data of three patients with different stages and patterns of keratoconus were assessed to develop a three-dimensional (3D) patient-specific finite-element model (FEM) of the keratoconic cornea. For each patient, the exact surgery procedure definitions were interpreted in the step-by-step FEM. Then, seven surgical scenarios, including different ICRS designs (complete and incomplete segment), with two surgical implementation methods (tunnel incision and lamellar pocket cut), were simulated. The pre- and postoperative predicted results of FEM were validated with the corresponding clinical data. For the pre- and postoperative results, the average error of 0.4% and 3.7% for the mean keratometry value ($\text {K}_{\text{mean}}$) were predicted. Furthermore, the difference in induced flattening effects was negligible for three ICRS types (KeraRing segment with arc-length of 355, 320, and two separate 160) of equal thickness. In contrast, the single and double progressive thickness of KeraRing 160 caused a significantly lower flattening effect compared to the same type with constant thickness. The observations indicated that the greater the segment thickness and arc-length, the lower the induced mean keratometry values. While the application of the tunnel incision method resulted in a lower $\text {K}_{\text{mean}}$ value for moderate and advanced KC, the induced maximum Von Mises stress on the postoperative cornea exceeded the induced maximum stress on the cornea more than two to five times compared to the pocket incision and the preoperative state of the cornea. In particular, an asymmetric regional Von Mises stress on the corneal surface was generated with a progressive ICRS thickness. These findings could be an early biomechanical sign for a later corneal instability and ICRS migration. The developed methodology provided a platform to personalize ICRS refractive surgery with regard to the patient’s keratoconus stage in order to facilitate the efficiency and biomechanical stability of the surgery.

## Introduction

Keratoconus is a progressive, non-inflammatory corneal disease, followed by thinning and protrusion of the cornea. The progression of the keratoconus can lead to visual problems by inducing irregular astigmatism, myopia, and corneal scars^1^. To date, different treatment options for keratoconus management have been examined depending on the severity of the disease. These clinical options are presented to improve the visual acuity of patients and to prevent the progression of the disease by stabilizing the corneal structure. The clinically evaluated approaches range from cross-linking^2^, implantation of intracorneal ring segments (ICRS)^3^, and lamellar keratoplasty^4^.

Clinical studies have shown that the implantation of ICRS is an effective method of controlling the progression of the disease and enhancing visual acuity^5^. ICRS are designed to be implanted into the corneal stroma, to reduce geometric steeping by inducing an arc-shortening effect. MyoRing (DIOPTEX)^6^ and KeraRing (Mediphacos)^7^ are two examples of complete continuous and discontinuous ICRS implanted into the stroma via an induced pocket and tunnel, respectively. In clinical practice, the choice of ICRS type depends mainly on the stage of keratoconus, the topographic pattern, and the surgical nomograms^8^. Despite the existence of practical nomograms, which provide suitable platforms for the selection of ICRS in different case scenarios, there are complications associated with the postoperative visual outcomes^9^. Immigration of ICRS, corneal melting, and extrusion of ICRS are among the most common postoperative complications.

From a mechanical point of view, the extrusion of ICRS is associated with locally induced stresses at the implant insertion site, which may be followed later by corneal melting and vascularization^10^. During postoperative implant extrusion, the cornea gradually weakens, leading to biomechanical instability and tissue failure. Thus, a robust assessment of the biomechanical changes induced by different types of ICRS in keratoconic eyes can optimize and improve the safety and efficiency of the surgery. At the same time, the stage and the pattern of the patient’s keratoconus have a direct influence on the upcoming surgical outcome. Therefore, the assessment of the visual contribution of both the biomechanical state of the patient’s cornea and the ICRS design is of great importance for surgical planning. With recent developments in numerical modeling, the performance analysis of refractive operations using the finite-element model (FEM) has become a popular field of research. Nevertheless, the use of FEM in corneal refractive surgery has led to a wide range of research activities, including numerical modeling of the laser in situ keratomileus (LASIK), implantation of ICRS, keratoplasty^11–17^.

Bao et al.^18^ developed the three-dimensional (3D) FEM of the LASIK procedure with regard to its effects on the biomechanical behavior of the cornea. They found that the agreement between the predictions of the numerical model and the clinical measurements significantly improved by considering the effects of the procedure on the biomechanical behavior of the cornea. Similarly, Fung et al.^19^ have investigated the biomechanical effects induced by wavefront aberrations after conventional refractive laser surgery. They showed that changes in corneal morphology induced by postoperative biomechanical effects are one of the factors influencing residual wavefront aberrations. In the keratoplasty studies, Canovetti et al.^20^ designed a two- and three-dimensional finite-element model of the cornea to evaluate the biomechanical loading capacity of different surgical wound configurations in penetrating keratoplasty. Later, Mohamed et al.^21^ investigated the stress and deformation of corneal tissue and donor graft during endothelial keratoplasty using a three-dimensional nonlinear FEM.

Lago et al.^22^ presented the first patient-specific FEM of refractive ICRS surgery using the isotropic hyperelastic model and validated the simulated radius of curvature with post-surgery outcomes. However, there were two main limitations in their study: First, the ICRS insertion was simulated by shifting the nodes of the pre-excitation hole instead of considering the realistic interaction between the cornea and the ICRS. Secondly, keratoconus is generally defined by the local morphological change that follows regional thickness reduction and corneal protrusion^23, 24^. Therefore, FEMs adopted anisotropic material models are the most representative models to define keratoconic eyes.

In another recent study, Flecha-Lescún et al.^25^ simulated ICRS insertion in axisymmetric FEM with mean corneal dimensions and isotropic hyperelastic material. Their work represents remarkable research by evaluating the influence of ICRS variables and insertion depth on the optical results. However, the contribution of the patient-specific anisotropic corneal model, as well as the asymmetric biomechanical effects induced by the contact interaction of the cornea-ICRS, was neglected.

To the best of the authors’ knowledge, the present paper is the first study to provide stepwise definitions of two main ICRS implementation methods, tunnel, and pocket incision, in a patient-specific anisotropic model of the cornea. This study numerically accounts for the asymmetric 3D effects of ICRS repositioning in the cornea to restore its equilibrium configuration. In particular, the repositioning mechanism of ICRS and cornea are considered to evaluate the surgically induced deformations, stresses, and strains. Furthermore, the influence of different ICRS types and implementation procedures on the postoperative biomechanical state and keratometry data in three individual keratoconus stages are investigated and validated. Finally, the following clinical questions are answered numerically; whether each ICRS design follows a similar trend of visual acuity improvement and stability in different keratoconus stages. At the same time, it is examined whether the same ICRS type with the variant implementation procedure induces a similar keratometry and biomechanical change. Although it is not possible to perform several surgical techniques on a single patient, the developed FEM represents a straightforward approach to evaluate different surgical scenarios.

## Results

A patient-specific finite-element model of two ICRS implantation procedures with seven individual surgical scenarios was developed for each keratoconus patient. Seven surgical plans were evaluated, including:

- MyoRing was implemented by the pocket cut method.
- KeraRing with the arc-length of 355 was implemented by the pocket cut method.
- KeraRing with the arc-length of 355, 320, and two separate segments with 160 arc-length were inserted by the tunnel incision method.
- KeraRing using one progressive thickness segment with a 160 arc-length, and two progressive segments with a 160 arc-length, were inserted by tunnel incision method.

All surgical scenarios were simulated for each participated patient. Based on the clinical tomographic data, patients were divided into three keratoconus patterns described as symmetric bow-tie (case one), oval (case two), and pellucid-like (case three). According to the AmslerKrumeich scales reviwed in^26, 27^ and the mean keratometry values of the patients, cases one, two, and three were categorized into grade three (advanced), grade one (mild), and grade two (moderate) of the disease. In this paper, by assigning the same ICRS characteristics (diameter, thickness) to all surgical strategies for each patient (Table 6), a comparative study was presented. The determined Von Mises stresses and strains were investigated as a promising method for biomechanical evaluation of postoperative configurations. The clinical significance of postoperatively induced stresses lies in their integration into later corneal stability states and in their long-term effects on corneal remodeling.

### Influence of corneal ICRS surgery on the biomechanical changes in three different stages of keratoconus

**Figure 1:**
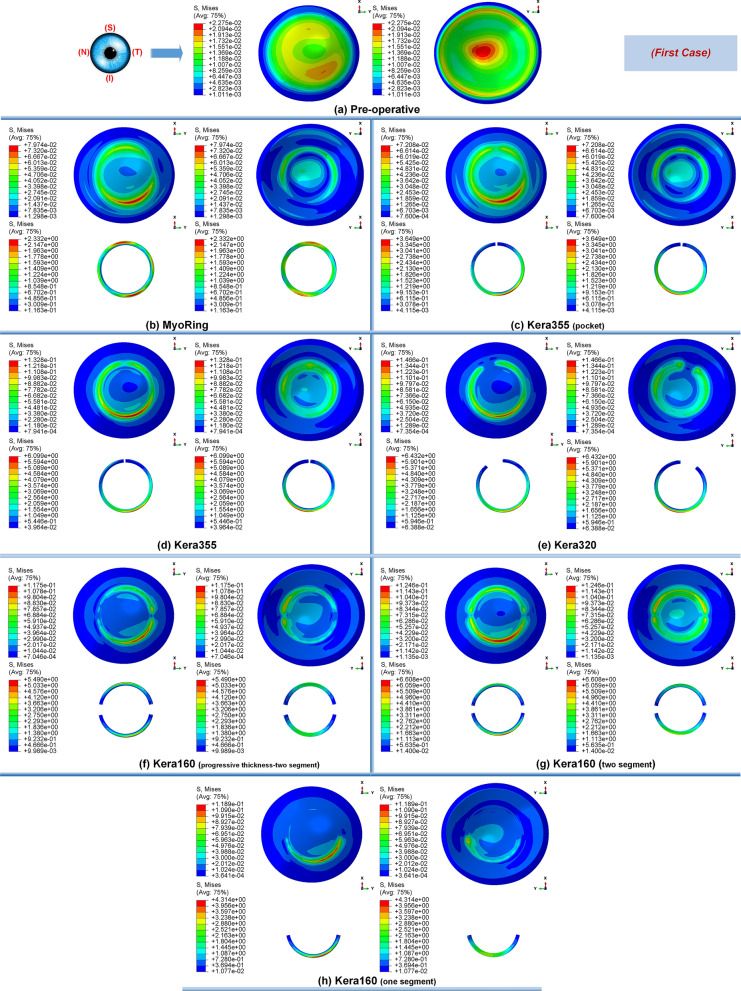
The pre- and postoperative contour diagrams of the Von Mises stress distribution (MPa) in FEM of different surgical scenarios (first case): (**a**) preoperative; (**b**) MyoRing; (**c**) Kera355 implemented by pocket incision; (**d**) Kera355; (**e**) Kera320; (**f**) Kera160-two prog segments; (**g**) Kera160-two segments; (**h**) Kera160-one prog segment.

Primarily, the different stages of the disease showed the same tendency towards biomechanical changes during the implementation operations of the ICRS. Preoperatively, each grade of keratoconus had a direct linear correlation with the state of corneal stresses. The more advanced the stage of keratoconus became, the greater the observed maximum Von Mises stresses, as shown in Figs. 1a, 12a, and 14a. In all three cases of keratoconus, the preoperative Von Mises maximum stress was between 17.78 kPa and 22.75 kPa, which was located on the anterior corneal surface below the nasal-temporal axis. At the same time, the position of maximum stress was in agreement with the site of maximum tangential curvature, shown in tangential maps (preoperative maps in Fig. 6). While in the preoperative states, the magnitude of the maximum Von Mises stresses correlated directly with the severity of the patient’s keratoconus, the postoperatively induced stresses followed a different trend. Postoperatively, the central keratoconus (case one and case two) experienced higher Von Mises stresses compared to the paracentral keratoconus (case three, where a higher preoperative stress state prevailed). This can be explained by the fact that the central Keratoconus put the ICRS in a state of greater compression and strain to achieve the central flattening effect, which exerts more tension on the cornea.

**Figure 2:**
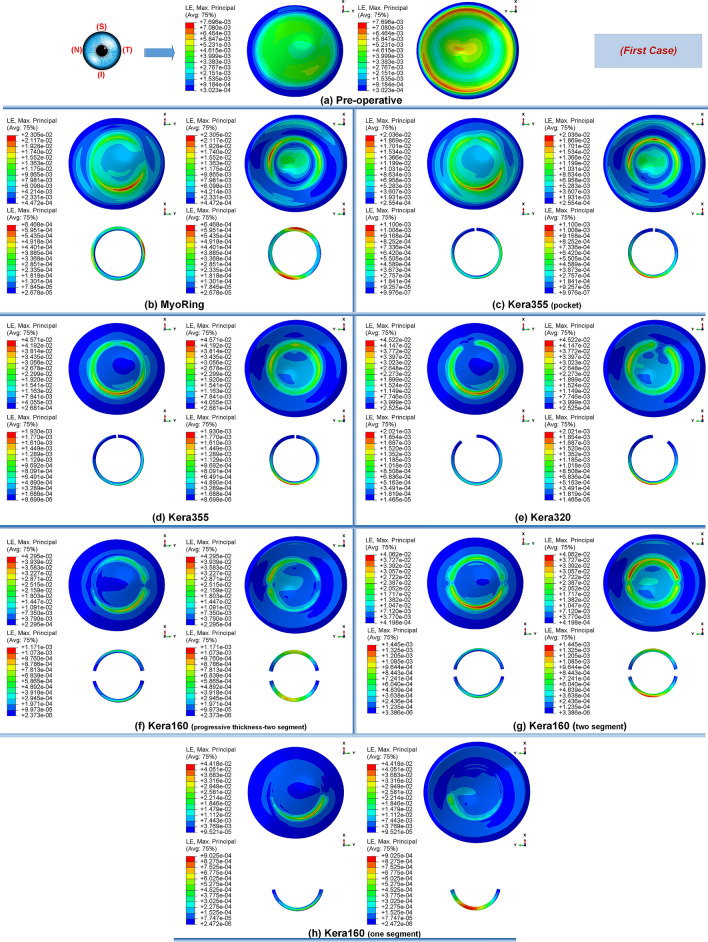
The pre- and postoperative contour plots of the principal strain’s distribution in FEM of the different surgical scenarios (first case): (**a**) preoperative; (**b**) MyoRing; (**c**) Kera355 implemented by pocket incision; (**d**) Kera355; (**e**) Kera320; (**f**) Kera160-two prog segments; (**g**) Kera160-two segments; (**h**) Kera160-one prog segment.

**Figure 3:**
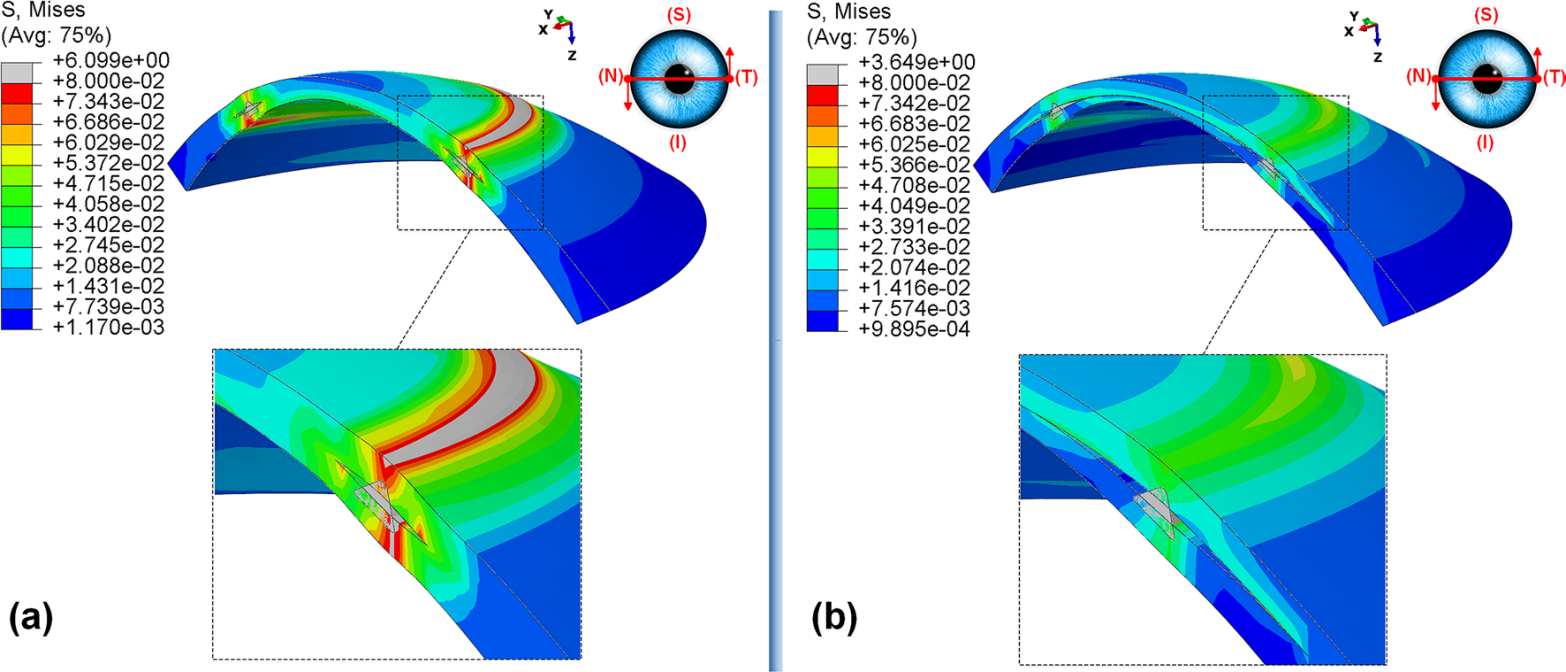
The postoperative contour of the Von Mises stress distribution (MPa) in the nasal-temporal cross-section of FEM of Kera355 implantation using (**a**) tunnel incision; (**b**) lamellar pocket cut (case one).

**Figure 4:**
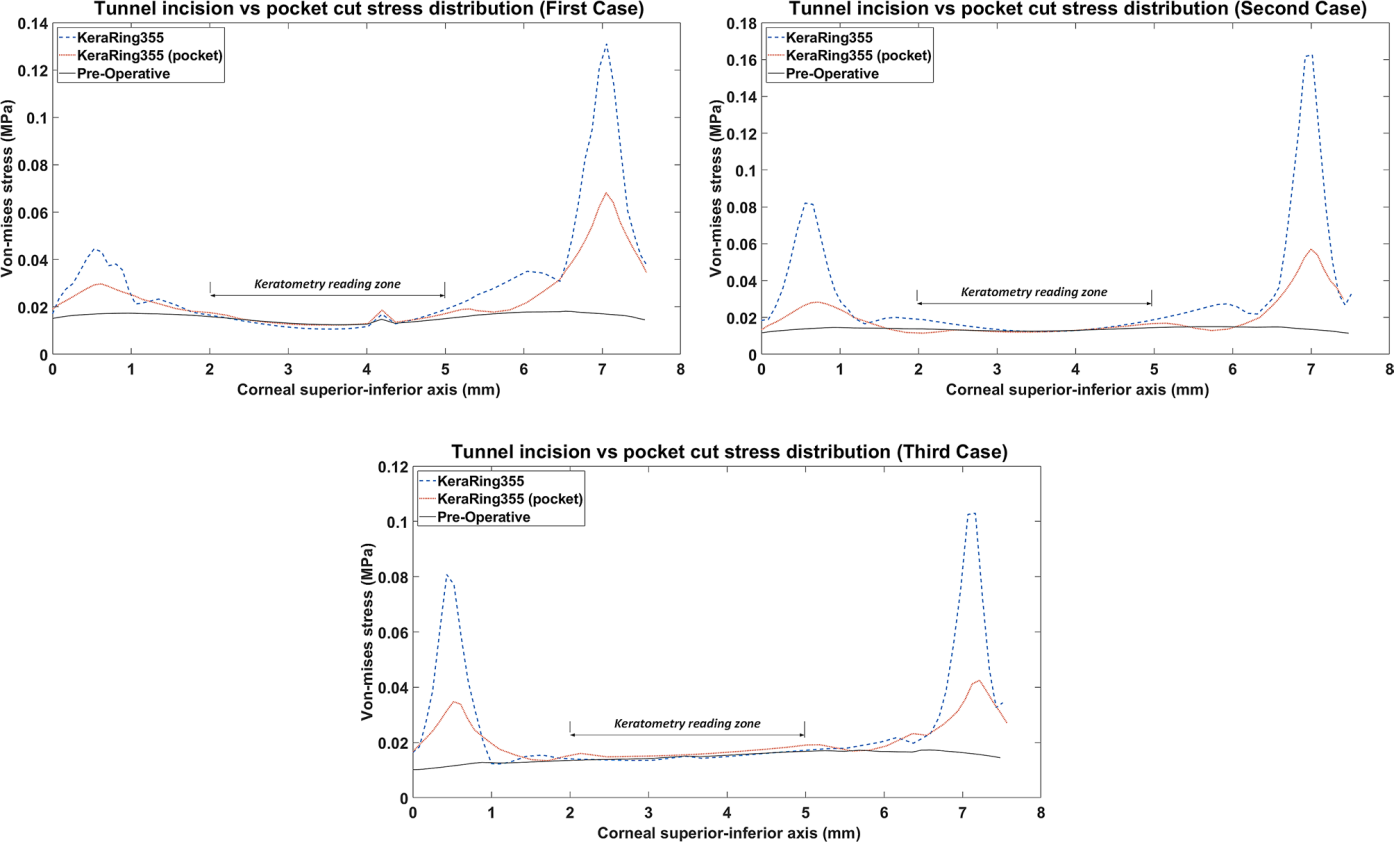
2D comparison plot of Von Mises stress distribution in superior-inferior direction on the anterior corneal surface for all case scenario. (Kera355 implemented by tunnel incision and pocket incision are shown in the figure by light blue (dashed-line) and red (dotted-line) respectively).

Secondly, the postoperative results showed that ICRS implantation generally produced an asymmetric circular shape with maximum Von Mises stresses and principal strains on the anterior corneal surface at the implant position. For all cases, the post-operated cornea experienced the highest Von Mises stress and principal strain in the inferior-superior direction on average in the range of 46.62 kPa to 169.6 kPa and 2.036e−2 to 6.29e−2, respectively. Similarly, the maximum Von Mises stresses and principal strains of the ICRS were in the range of 1.67 MPa to 7.02 MPa (Figs. 1, 12, and 14) and 2.45e−4 to 2.02e−3 (Figs. 2, 13, and 15), which were observed in the inferior-superior direction. In practice, the greatest curvature steeping in the inferior-superior direction in a pre-operated cornea resulted in the highest tension on the ICRS being flattened and, conversely, this produced the maximized stresses and strains on the operated cornea in the same direction.

Thirdly, in the postoperative evaluation, the maximum Von Mises stresses reached the highest value in the first case, while the strain showed the highest value in the third case. In the first case, stress was on average 30% to 40% greater than in the third case. However, the assignment of a higher matrix stiffness (c10 with an amount 50% higher than in the third case) resulted in a lower induced principle strain on the cornea. Interestingly, this emphasized the fact that the morphological condition and strength of the patient’s cornea also plays a significant role in the postoperative biomechanical state and stability. The posterior corneal surface in the central cornea (5 mm diameter) showed higher maximum stress and strain than the anterior corneal surface in all case scenarios. Biomechanically, this can be explained by the fact that the placement of ICRS at 70% depth, relative to the clinical intervention, caused higher stresses and strains on a surface near the ICRS than on the distant surface. In contrast, at the implantation position (5 mm to 7 mm diameter), greater stresses were induced on the anterior corneal surface due to the cross-sectional shape of the ICRS, which was penetrated to interlamellar. In fact, the lower ICRS surface runs along the posterior interlamellar corneal surface, while the upper ICRS surface creates a normal force on the anterior interlamellar corneal surface, resulting in greater tension and elongation at the implantation position on the anterior corneal surface.

### Influence of ICRS implementation procedures on the corneal biomechanics and stability in three stages of keratoconus

In this study, two methods of ICRS implementation, the lamellar pocket cut, and the tunnel cut were investigated using the same ICRS types (Kera355). The results show that the methods induce two different levels of stress on the postoperative cornea (Fig. 1c,d). The examination of all three cases suggests that the highest Von Mises stress in a cornea operated by tunnel incision reaches an order of magnitude of 81.32 kPa to 169.6 kPa, while the similar operation with pocket incision induces much lower stress of 46.92 kPa to 79.74 kPa. In particular, in the first case, the ectatic cornea experiences a maximum Von Mises stress of 132.8 kPa after the tunnel incision method (Fig. 1d), while a similar operation with the pocket incision method (Fig. 1c) induces a much lower maximum stress value of 72.08 kPa on the operated cornea.

The observation of the principal strain also shows a much lower induced strain in the lamella pocket compared to the tunnel incision method. The principal strain during tunnel incision induced elongation of 4.57e−2 (Fig. 2d), while this value caused an elongation of about 2.036e−2 during pocket incision (Fig. 2c). At the same time, the ICRS experienced a similar trend in maximum Von Mises stresses in two implementation methods. The maximum Von Mises stresses and principal strains generated in the ICRS by tunnel cutting methods were around 2 times higher than those generated by the pocket cutting method (Fig. 1c,d). The introduction of the ICRS via a tunnel incision forces the ICRs into a limited area defined by the inner and outer diameter of the tunnel, whereas with the lamellar pocket incision the ICRS has the chance to transfer their flattening pressure to the circular pocket area of the cornea.

The postoperative outcome of Kera355 implantation using the tunnel incision and lamellar pocket cut methods was compared in Fig. 3. The result shows the contour levels of the Von Mises stress distribution in the corneal cross-section along a nasal-temporal direction. It was evident that the two methods provide two different stress distribution patterns along the corneal thickness direction. While in the tunnel incision method, a gradual increase in Von Mises stress was observed from the anterior to the posterior surface, in the pocket method, two separate corneal layers act as single layers, with the upper layer being more stressed than the lower, resulting in a greater lamellar susceptibility to IOP. In addition, despite the consistent change in tension around the implant, a higher irregular variation in the maximum Von Mises stress on the cornea at the implant position was observed with the tunnel incision method.

Figure 4 shows the comparative analysis of 2D stress distribution on the anterior corneal surface in the inferior-superior direction. In this comparison, two different implementation methods using a Kera355 were compared with the preoperative stress distribution state. The result demonstrates that the tunnel incision method induces significantly higher maximum Von Mises stresses at the implementation site. Approximately, it presents more than 2 times higher stresses than the lamellar pocket cut and 5 times higher than the preoperative stress configuration. In the tunnel incision method, the peak of the Von Mises stress profiles, followed by a sudden decrease of the stress level in the central cornea, while a gradually declining in stress distribution through the pocket cut method was observed.

Finally, in the optical zone (5 mm), the postoperative stress distribution on the anterior corneal surface remained approximately unchanged with both methods. Despite differences in the magnitude of the maximum induced stress in the peripheral, mostly central reading zone of corneal keratometry (3 mm diameter), a similarly high Von Mises stress followed in both methods. In the first case and the third case, the tunnel incision induced a slightly lower value of Von Mises stress. While in the first implementation method, the center of the cornea was manipulated by a lamellar incision, in both methods the Von Mises stress in the reading zone of keratometry remained unaffected and biomechanically stable compared to the preoperative state.

Nevertheless, these considerable stress concentrations and fluctuations obtained for the tunnel incision method, illustrated in both qualitative (Fig. 3) and a quantitative (Fig. 4) evaluation, provide a biomechanical interpretation for the implant extrusion in the progression of ectasia. In particular, any provocation factor, such as eye rubbing and sudden eye impact, may increase the local maximum stress concentration, especially at the site of ICRS implantation, and lead to corneal failure in maintaining ICRS. Consequently, the tunnel incision method with a high level of inducing stress could be a risk factor for long-term stabilization of the surgical outcome and postoperative complications. In summary, although the effect of different ICRS features such as ICRS thickness, diameter, implementation depth, were investigated in various clinical and numerical studies, it should be noted that the keratoconus stage and implementation method also must be considered as an influencing factor on postoperative biomechanics and stability.

### Influence of ICRS design on the corneal biomechanics and stability

The induced maximum stresses and strains with different KeraRing types implemented by the tunnel incision method showed a variety of magnitude in post-operated corneas (Fig. 1). From Figs. 1, 2 it was seen that the maximum principal strain and the Von Mises stress on the anterior corneal surface mainly showed a circular stress concentration with symmetrical distribution for ICRS types, including Kera355, Kera320, and Kera160-two segments, while the post-operated cornea with Kera160-two prog segments and Kera160-one prog segment showed asymmetrical stress and strain distribution (Figs. 1, 2). Additionally, the types Kera355, Kera320, and Kera160-two segments showed higher induced stresses in comparison with progressive types. In progressive thickness types, the thickness decreased linearly by 0.1 mm in all three cases. Thus, the thickness and arc-length of the ICRS proved to be another influencing factor in the induction of stress and stability of the post-operated cornea.

**Figure 5:**
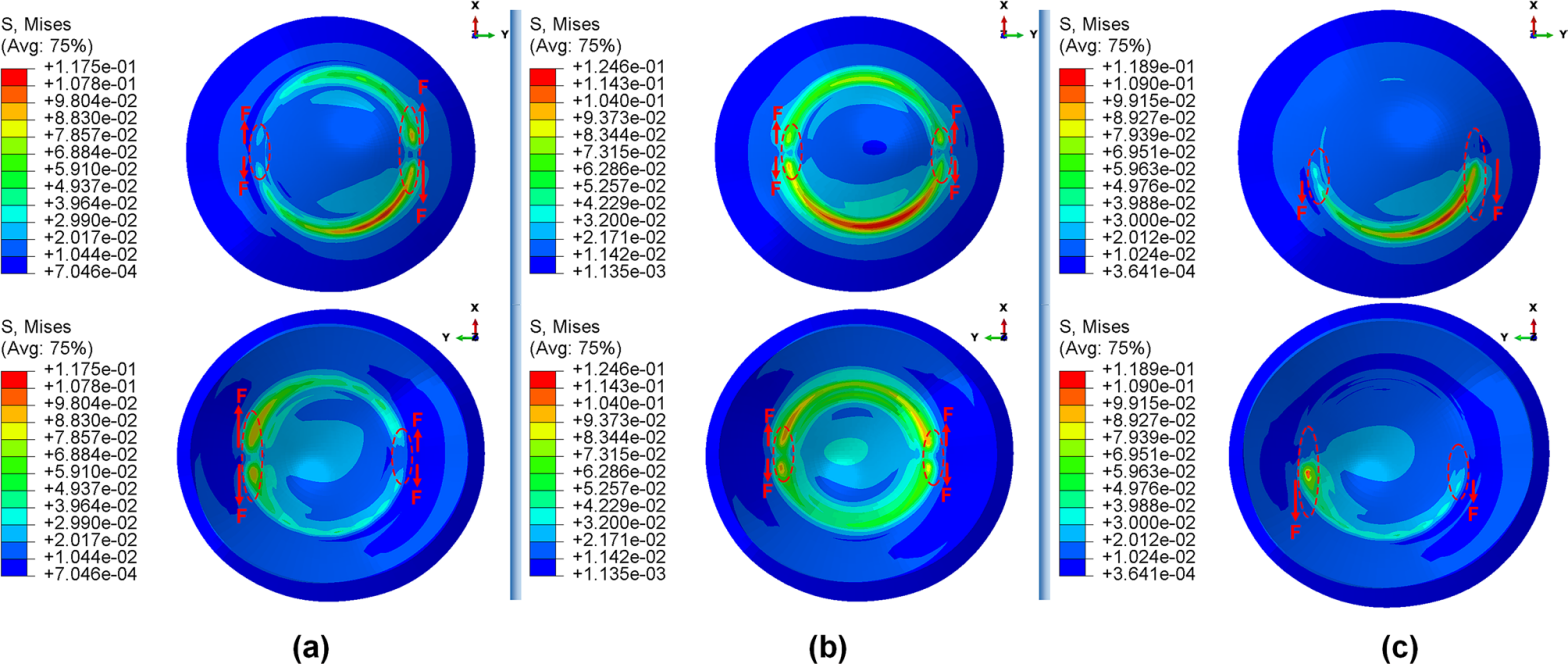
Comparison of the Von Mises stress distribution (MPa) and the contour of locally induced stresses at the ICRS endpoints for three different types of ICRS, including: (**a**) Kera160-two prog segments; (**b**) Kera160-two segments; (**c**) Kera160-one prog segment.

It is important to note that the final stability of the ICRS in the cornea also depends directly on the balance between the maximum locally induced forces and the torques on the cornea. Changes in these forces could create a biomechanical imbalance and increase the risk of implant failure. Given this fact, Fig. 5 compared the stress distribution of Kera160-two segments, Kera160-two prog segments, and Kera160-one prog segment as three types of incomplete ICRS. The results obtained for induced Von Mises stress on the corneal surface allow us to assess the differences between these three types. While the implantation of segments in a circular tunnel leads to a local stress build-up on the upper layer of the implant, in the incomplete implants the corneal layers endure the additional local stresses at the segment ends. Especially the complete segments by slightly squeezing could lead to a flattening of the cornea, the incomplete segments lead to local forces and tensions on the cornea due to their inconstant geometries.

As a result, the torque created by these local forces has been transmitted to the corneal flap and may cause the ICRS to reposition itself or the cornea to be reshaped to compensate stresses in a new configuration. These induced forces lead to a state of imbalance that was further exacerbated in the implementation of progressive types by inducing higher values at thicker ends of the implant. In addition, in the implementation of the Kera160-one prog segment at the ends of each segment in the lower half of the cornea, while the upper half of the cornea remains unaffected, leading to a biomechanical imbalance configuration. Thus, this observation also supports clinically reported cases of ICRS immigration. The progression of the tension in the position of the segments could significantly influence the surgery biomechanical stability. In other words, locally induces stresses could gradually alternate the balance of the ICRS-cornea and lead to the repositioning of ICRS and once the cornea loses its biomechanical equilibrium, the cornea melting could happen. These findings provide biomechanically clear perceptual insights of surgery stability.

### Influence of ICRS implementation procedures and design on the topographic map of the cornea and keratometry values (FEM verification)

To validate the result and to enable a comparative analysis in clinical terms, topographic maps and keratometry values were generated from all simulated surgical scenarios. The following tomographic data were evaluated: corneal dioptric power in the flattest meridian (K1), corneal dioptric power in the steepest meridian (K2), and mean corneal power (Km), where average Keratometric values for 3 mm pupil diameter, in diopters were derived. Primary, a mathematical description of 8th Zernike polynomial in the form of z = f(x,y) was fitted to the anterior corneal surface (corneal anterior curvature area(CACA) is shown in Fig. 9) of the derived FEM. Then, with the assumption of f(x,y) is twice differential, surface two invariants, named as the Gaussian curvature and mean curvature^28^, respectively obtained as:

1 $$\begin{aligned} \begin{aligned} K=\frac{f_{x x} f_{y y}-f_{x y}^{2}}{\left( 1+f_{x}^{2}+f_{y}^{2}\right) ^{2}} \end{aligned} \end{aligned}$$

2 $$\begin{aligned} \begin{aligned} H=\frac{\left( 1+f_{x}^{2}\right) f_{y y}-2 f_{x} f_{y} f_{x y}+\left( 1+f_{y}^{2}\right) f_{x x}}{2\left( 1+f_{x}^{2}+f_{y}^{2}\right) ^{3 / 2}} \end{aligned} \end{aligned}$$

**Figure 6:**
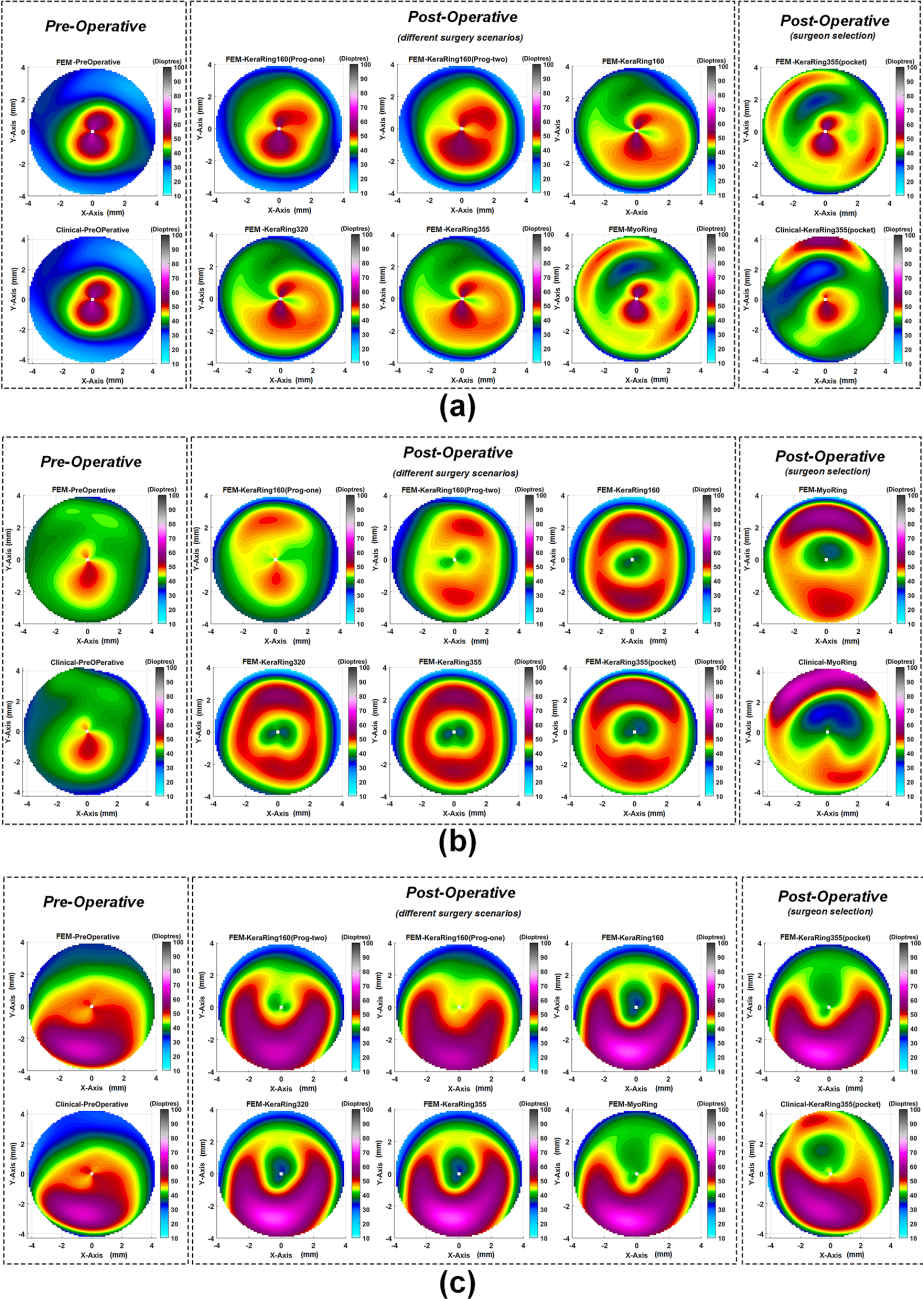
The pre- and postoperative maps of tangential curvature tomography (D) obtained at FEM from different surgical scenarios with respect to clinical reported maps for the (**a**) first case, (**b**) second case, (**c**) third case.

Secondly, the orthogonal principal curvatures of corneal anterior surface using Eqs. (1) and (2) calculated:

3 $$\begin{aligned} \begin{aligned} k_{1}=H-\sqrt{H^{2}-K}, \quad k_{2}=H+\sqrt{H^{2}-K}, \quad \end{aligned} \end{aligned}$$

where $n_{1}=1.3375$ is the standard Keratometric index (SKI) and $n_{0}=1.0$ represents the refractive index of air. For $h_{x},$ etc. given in real units, the curvatures $k_{1}, k_{2}$ and $F$ have units of reciprocal meters, while $K$ has units of reciprocal meters squared. FEM simulated keratometry values of steep (K1) and flat (K2) meridians were derived from pre- and postoperative geometries using Eq. (3). The average of the steep (K1) and the flat (K2) meridians was reported as $K_{mean}$. As previously elaborated, tangential curvature maps of the pre- and post-operated geometries for various ICRS design in three different case scenarios were plotted in Fig. 6.

The pre- and postoperative keratometry results of the developed FEM, for seven different operation scenarios, were determined. The corresponding keratometry outcome for each keratoconus cases were listed in Tables 1, 2, and 3. In moderate (case three) and sever (case one) form of keratoconus, the result indicates that the surgical scenarios with the tunnel incision method induce a further flattening effect on the central cornea in comparison to the lamellar pocket cut operations. From a biomechanical perspective, implementing a lamellar cut degrades the corneal consistency and stiffness by introducing two separates weaken and thinner tissue. Thus, these two tissues form a new individual equilibrium state, which is more exposable to IOP, resulting in further displacement and tension on central corneal comparing tunnel incision. As can be seen in Fig. 4, the application of the tunnel incision method in the first and third cases resulted in a lower Von Mises stresses in the central cornea (blue dashed line), which could lead to a higher flattening effect in the keratometry reading area.

**Table 1:** Pre- and postoperative keratometry results of the first case.

Type	$\text {K}_{\text{1}}\, (D)$	$\text {K}_{\text{2 }}\, (D)$	$\text {K}_{\text{mean }}\, (D)$	$\Delta \text {K}_{\text{mean }}\, (D)$	Error^a^ $(\%)$
Pre (FEM)	46.93	58.63	52.78	–	0.3
Pre (clinical)	46.91	58.33	52.62	–	–
Post-Kera160-one prog segment (FEM)	46.25	54.62	50.4	− 1.46	5.74
Post-Kera160-two prog segments (FEM)	45.86	52.88	49.23	− 2.63	3.29
Post-Kera160-two segments (FEM)	42.02	53.49	47.75	− 4.11	0.18
Post-Kera320 (FEM)	41.99	53.42	47.7	− 4.16	0.083
Post-Kera355 (FEM)	41.99	53.42	47.7	− 4.16	0.083
Post-MyoRing (FEM)	43.44	55.32	49.38	− 2.48	3.6
Post-Kera355-pocket (FEM)	43.49	55.4	49.45	− 2.41	3.75
Post-Kera355-pocket (clinical-1month)	42.91	49.47	46.16	− 5.43	–
Post-Kera355-pocket (clinical-3month)	44.04	51.29	47.66	− 4.96	–

^a^Percentage error of FEM predictaed $\text {K}_{\text{mean}}$.

**Table 2:** Pre- and postoperative keratometry results of the second case.

Type	$\text {K}_{\text{1}} \, (D)$	$\text {K}_{\text{2 }}\, (D)$	$\text {K}_{\text{mean }} \, (D)$	$\Delta \text {K}_{\text{mean }} \, (D)$	Error^a^ $(\%)$
Pre (FEM)	42.63	48.32	45.48	–	0.73
Pre (clinical)	42.42	47.87	45.15	–	–
Post-Kera160-one prog segment (FEM)	42.67	46.95	44.81	− 0.9	15.27
Post-Kera160-two prog segments (FEM)	41.53	45.66	43.6	− 2.71	12.74
Post-Kera160-two segments (FEM)	39.49	44.05	41.77	− 4	8.01
Post-Kera320 (FEM)	38.5	44.25	41.38	− 4.33	7
Post-Kera355 (FEM)	38.27	43.93	41.10	− 4.61	6.28
Post-MyoRing (FEM)	38.35	41.89	40.12	− 5.59	3.7
Post-Kera355-pocket (FEM)	37.5	42.16	39.87	− 5.84	3.1
Post-MyoRing (clinical-6month)	36.36	40.99	38.67	− 6.48	-

^a^Percentage error of FEM predictaed $\text {K}_{\text{mean}}$.

**Table 3:** Pre- and postoperative keratometry results of the third case.

Type	$\text {K}_{\text{1}}(D)$	$\text {K}_{\text{2 }}(D)$	$\text {K}_{\text{mean }}(D)$	$\Delta \text {K}_{\text{mean }}(D)$	Error^a^ $(\%)$
Pre (FEM)	47.07	50.18	48.62	–	0.26
Pre (clinical)	46.73	50.78	48.75	–	–
Post-Kera160-one prog segment (FEM)	44.6	47.13	45.87	− 1.68	1.3
Post-Kera160-two prog segments (FEM)	42.43	46.77	44.6	− 2.95	1.3
Post-Kera160-two segments (FEM)	37.6	43.68	40.6 4	− 6.91	10.24
Post-Kera320 (FEM)	40.61	43.65	40.61	− 6.94	10.34
Post-Kera355 (FEM)	37.56	43.65	40.6	− 6.95	10.31
Post-MyoRing (FEM)	41.49	46.18	43.84	− 3.71	3.18
Post-Kera355-pocket (FEM)	41.34	46.19	43.76	− 3.79	3.35
Post-Kera355-pocket (clinical-8month)	42.98	47.58	45.28	− 3.47	–

^a^Percentage error of FEM predictaed $\text {K}_{\text{mean}}$.

On the contrary, in the second case (a mild form of keratoconus) with a higher minimum thickness (Table 4), the introduction of a lamellar incision had no significant effect on the central corneal area tensions, as shown in Fig. 4 (the red (dotted-line) and blue (dashed-line) lines were relatively close together and follow approximately the same path). As a result, the outcome of keratometry shows no significant difference between the two methods.To evaluate the result in more detail, Kera355, Kera320, and Kera160 (two segments) presented similar topographic maps and postoperative keratometry outcomes. Meanwhile, the topographic map in Kera160 (two prog segments) and Kera160 (one prog segment) showed no significant improvements in the central cornea and remained mainly unchanged. These two types provide a less flattening effect compared to others. As a result, the greater the ICRS thickness and longer arc length, the higher reduction in the average K reading were achieved. This statement is in agreement with previous clinical studies^29, 30^. Furthermore, similar improvements in keratometry results between Kera355 and the MyoRing employing pocket cut method and a similar topographic pattern, in all three cases were observed, as shown in Fig 6, and Tables 1, 2, and 3. In the validation procedure, the result of the predicted keratometry values from the developed FEM was compared with clinical data of the same surgical scenario for each patient. Consequently, $\text {K}_{\text{mean}}$ were obtained from the anterior surfaces of both the actual topographies and their generated FEMs, at pre- and post-surgery intervals. Prediction error in $\text {K}_{\text{mean}}$ were calculated according to difference in actual and simulated ICRS-induced changes, which were reported in percentage. The obtained preoperative error percentage of $\text {K}_{\text{mean}}$, for all cases, was an average of 0.4%.

The individual comparisons of these differences were listed in Tables 1, 2, and 3, support that preoperative FEM prediction values were accurate and within the device accuracy of 0.5D. Moreover, The postoperative prediction error in $\text {K}_{\text{mean}}$ for each three patient, including case one, case two, and case three, were 3.75%, 3.7%, and 3.35%, respectively. For case one, the surgically induced changes in $\text {K}_{\text{mean}}$ with the numerical simulations were compared to the actual values from postoperative topography measurements obtained 1-month, and 3-month post-surgery (Table 1). Interestingly, the accuracy of the FEM predictions was higher for the measurements taken 3-months (the reported error was 3.75% ) rather than the 1-month (the reported error was 7.12%) after the intervention. In conclusion, the average prediction error was about 3.6%. Although there was no statistically significant error, it was above the instrument accuracy of 0.5 D. As mentioned in previous studies^11^, the rotation and misalignments errors may explain the outliers in the numerical predictions, as well as the limited access to an objective metric for the alignment of the pre- and postoperative topography data.

The corneal misalignment (rotation, tilt, and decentration) is one the profound source of predicted error in the estimation of optical and keratometry result in keratoconic eye^31^. Indeed, misalignment induced errors demonstrate the direct correlation with keratoconus severity^32^. Accordingly, individual fluctuations of keratoconus corneal curvature can exceed the repeatability of the corneal tomographer. Aside from that, the corneal remodeling and recovering its stable configuration after ICRS refractive surgery, which for each patient due to its different corneal pathology, follows a unique and unknown trend, causing another source of unpredicted error. Meanwhile, the unavailability of the representative constitutive equation defining the keratoconus pathology with patient-specific material constant along with other reason, all relatively affected the FEM predicted outcomes. The investigated computational modeling approach demonstrated low prediction errors and may be useful for clinical guidance in planning ICRS implementation surgery.

## Discussion

The ICRS implementation surgery is an effective corneal refractive surgery for keratoconus management. Although recent developments in ICRS refractive surgery have significantly reduced the incidence of postoperative complications, such as ICRS extrusion and immigration, the factors that influence the postoperative accuracy and safety are still poorly understood. Moreover, the surgical results can be directly influenced by the preoperative biomechanical factor, such as the ICRS design, the implementation procedure, and the keratoconus stage of the patient. Therefore, understanding the effects of various factors that influence the biomechanical conditions of the cornea will help to determine the best treatment option and improve the postoperative stability and safety of the surgery. Thus, the present methodology numerically investigated the minimally invasive surgical procedure for keratoconus treatment with the best keratometry results.

The $\text {K}_{\text{mean}}$ differences between the predictions of FEM and the clinical results in the preoperative evaluation, derived for all cases with an average error rate of 0.4%. However, the postoperative results showed that FEM was able to predict the clinical keratometry outcome with an error on the induced $\text {K}_{\text{mean}}$ of 3.75%, 3.7%, and 3.35% for cases one, two, and three. Two important sources of prediction errors in the present simulations are the high probability of device misalignment in keratoconus cases and corneal remodeling. Furthermore, the Inaccessibility of precise patient-specific material properties for keratoconus patients leads to further uncertainty of results. The keratometry results demonstrate that the tunnel incision method generally showed a greater flattening effect in a moderate and advanced form of keratoconus compared to the pocket incision method. The induction of a lamellar incision in the central cornea resulted in two layers of morphologically and geometrically weakened features that caused greater elongation and local curvature in response to IOP, especially in the progressive form of the keratoconus. With regard to these biomechanical observations, the average keratometry shows a similar finding by induction of a lower $\text {K}_{\text{mean}}$ in a pocket incision compared to the tunnel incision in case one (advanced) and case three (moderate). Moreover, in evaluating different types implemented by the tunnel incision method, the KeraRing segments with progressive thickness, provide a considerably lower flattening effect in comparison to others, which demonstrate the direct influence of higher thickness and longer arc length, to decrease the average keratometry result.

The developed FEM of ICRS implementation operation was used to evaluate the stress, and strain distributions on the post-operated cornea, in different ICR case scenarios. The first main result was the observed difference in the implementation methods; the tunnel cut method caused a significant amount of maximum Von Mises stress at the implementation site compared to the lamellar pocket cut method. The second FEM evidence shows that while the first case with the preoperative highest keratometry value and thinnest minimum corneal thickness resulted in a higher postoperative Von Mises stress compared to the third case, the assignment of a higher matrix stiffness (c10 with a 50% higher value compared to the third case) resulted in a lower induced principal strain in the first case. This finding is consistent with the postoperative keratometry results.

The comparison of the keratometry results of the Kera355 (implemented by tunnel incision method) in these two cases shows that while in the third case, a central flattening occurred with an average value of − 6.9 D, a similar surgical scenario with a higher material stiffness of the patient caused an average flattening of − 4.14 D, in the first case. The result showed the necessity to consider the influence of morphological characteristics of keratoconus patients on the selection of surgical scenarios. In the third evident, the results of the FEM found irregular Von Mises stress concentration, with incomplete segments which could lead to imbalance local forces and torque, as shown in the Fig. 5 These local stresses could be another risk factor for later spontaneous focal thinning, which occurs at the segment incision site on the corneal stroma.

The depth and thickness of ICR implementations in these procedures could act as critical factors in increasing these local stresses. The closer the ICRS were implemented to the corneal surface, and the ticker it became, the higher the stress concentration was induced. From a technical point of view, local stresses generated during implant migration could accelerate corneal weakening and cause implant extrusion. Clinically, investigated by Mounir et al.^10^, the prevalence and risk factors of ICRS extrusion were performed by femtosecond laser. They found that careful evaluation and timing of the CXL can be considered as preoperative options to achieve satisfactory results. In an earlier study^33^, they reported the incidence of complications for manual interstation at $18.11 \%$, with spontaneous extrusion of the ICRS occurring at $5.66 \%$, while with the femtosecond laser only $3.6 \%$ of the eyes affected by implant extrusion occurred. Accordingly, these observations clinically support the biomechanically assessed influences of corneal stiffness and implementation technique on corneal instability and implant extrusion.

Nevertheless, in the present work, several limitations should be considered. Firstly, further studies with larger samples, comprising more cases in each keratoconus pattern and stage and repeated longer follow-up periods, should be considered to be representative for the entire population. However, the validity and comparative nature of the present results support the conclusive observations of the study. Secondly, the corneal stiffness and local corneal microstructure have a significant impact on postoperative outcomes. Thus, although it was realistically assumed that corneal tissue is hyperelastic and anisotropic, a representative constitutive equation that considers the pathology and microstructure of keratoconus with patient-specific constants also need to be evaluated. Thirdly, in the current study, the effects of fluid-structure interaction between the cornea and the inner fluid of the eye were neglected due to the high computational effort and the allocation of more uncertainty. However, it is recommended to examine the influence of the interaction of the inner fluid on the final equilibrium state of ICRS-cornea in future studies. Finally, patient-specific ray-tracing analysis concerning the axial length of the patient’s eye and other features to evaluate the contribution of a surgical factor to postoperative optical parameters will be an added advantage compared to the keratometry result.

The main contributions of the present work can be listed as follows: To the best of the author’s knowledge, this work is primarily the first study in which two main techniques of ICRS implementation were numerically evaluated in a patient-specific FEM by considering the interaction of the ICRS with the intracorneal lamellar and stepwise surgical scenario in finite-element definitions. Secondly, seven different ICRS surgical scenarios for three stages and patterns of keratoconus cases were investigated and clinically validated by simulating the results of topographic maps and keratometry data. Finally, the postoperatively induced stress and strain configuration was used to analyze the risk factors that influence corneal stability and progression management in different ICR designs. Given the novelty of this method, there were few clinical studies on the influencing factor that support the safety and stability of ICRS.

In conclusion, this paper presents a patient-specific finite-element model of two ICRS implementation methods, including tunnel incision and pocket cut. The developed FEM was investigated for seven different surgical scenarios of a complete and incomplete ICRS at three different degrees of keratoconus. The comparison of two implementation methods for Kera355 indicated that although a similar level of stress was observed in the central cornea with both methods, more than twice higher maximum stresses were found at the ICRS implementation site for the tunnel incision method. In fact, the sudden change in the stress level on the postoperative cornea could be a biomechanical explanation for the migration and subsequent extrusion of the ICRS. Compared to the pocket incision method, the tunnel incision method has a greater flattening effect, especially in advanced keratoconus. Biomechanically, it can be explained that the performance of a pocket incision resulted in two weaker and thinner layers, which could be more strongly influenced by the IOP. The evaluation of the KeraRing with progressive thickness implied the importance of the ICRS thickness and the arc-length of the result obtained in keratometry. Additionally, the segments with progressive thickness produced an asymmetric stress concentration on the cornea, which could lead to another cause of corneal instability. The developed methodology predicted clinical results with less than 4% error in keratometry flattening values. The results support the accuracy and efficiency of the developed FEM, which enables supervised surgical planning and facilitates the prediction of surgical safety and reliability.

## Concluding remarks

The results presented here demonstrate that in the procedure of ICRS implementation, the employment of the pocket cut technique led to an approximately uniform stress distribution over the inner layers of the post-operated cornea, while the tunnel incision method induced a high fluctuation of tensions. In particular, in the advanced form of keratoconus, high induction of stress concentrations could account for early signs of ICRS later instability and immigration. This study suggests that the employment of a complete form of ICRS would be a biomechanically more reliable option comparing to partial segments, given the considerable amounts of local stresses induced on the cornea at the segment endings.

Comparison of different types of incomplete ICRS, including the KeraRing segment with arc-length of 355, 320, and two separate 160 using the same implementation method, did not show significant differences in their induced flattening effects, while a considerably lower mean keratometry values were observed for single segments scenarios. Moreover, the implementation of a specific ICRS with each method demonstrated a particular shape regularization trend for different stages and patterns of keratoconus. These findings further emphasize the importance of customized computational modeling for ICRS refractive surgical planning.

## Materials and methods

**Figure 7:**
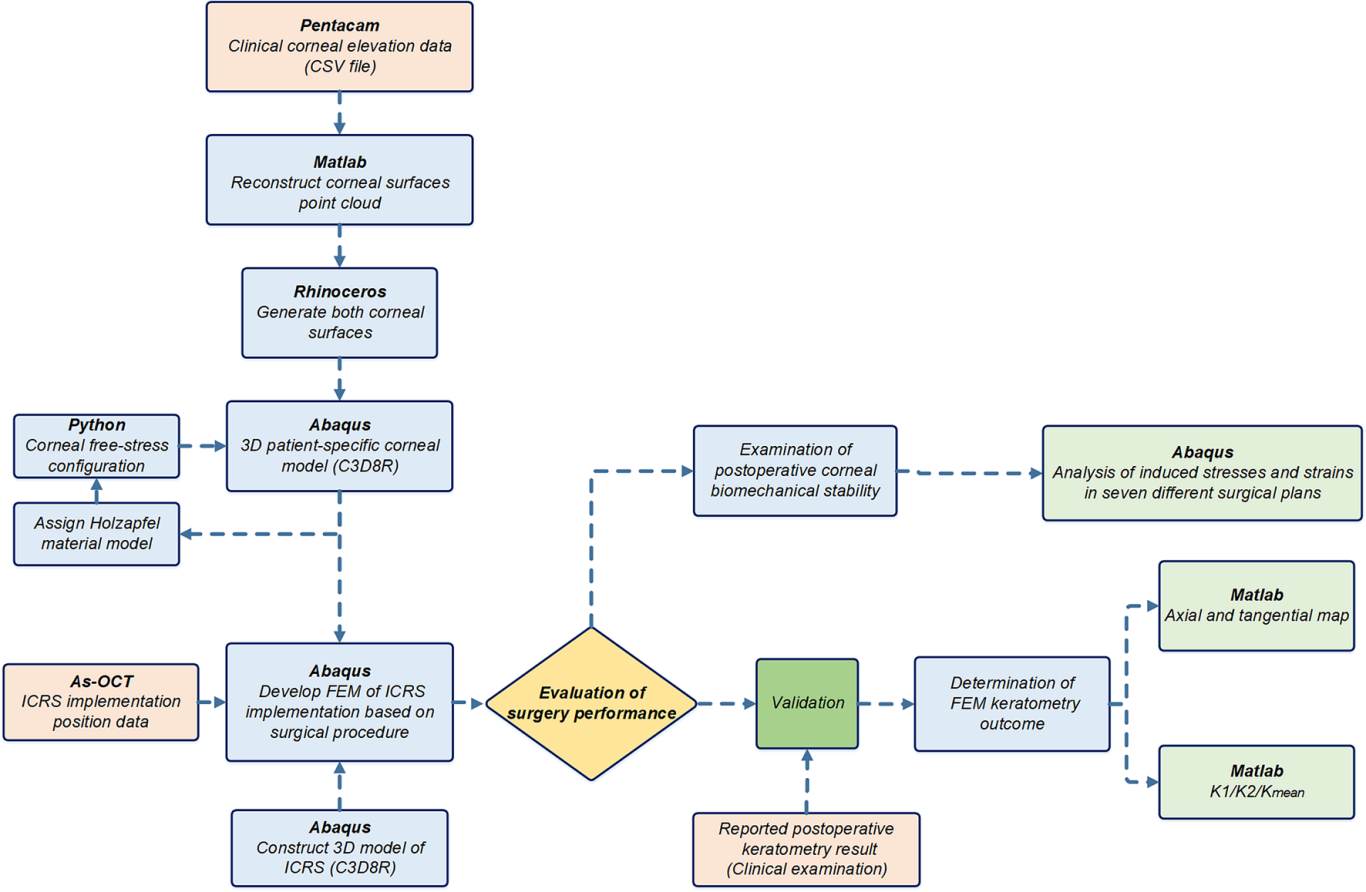
The schematic flowchart of the steps used in the development and evaluation of the patient-specific FEM of ICRS implementation surgery.

The development of a patient-specific numerical model for refractive surgery usually involves three main steps, including the construction of the cornea from clinical data, the definition of the surgical procedure from clinical practice in finite-element definitions, and validation. In this context, the following sections present the steps used in the development and evaluation of patient-specific simulation of ICRS implementation surgery (Fig. 7).

### Patients data collection

In the current research, three keratoconus corneas of three patients were included, each representing a specific keratoconus pattern (symmetric bow-tie, oval, pellucid-like) and belonging to three different disease stages. This study was approved by the Institutional Review Board at Isfahan University of Technology and conducted in accordance with the tenets of Declaration of Helsinki. Informed consent was obtained from all participants. One of the commonly used maps in topographic measurements is the axial map, which measures the distance from any point on the corneal surface that is on the sagittal plane from the visual axis of the cornea. This map is mainly used as a clinical guide to assess the overall shape and pattern of the cornea. Nevertheless, axial or sagittal topography maps are very sensitive to tilt and are not fully representative of the actual eye shape, especially in patients with keratoconus. Therefore, tangential or meridional maps are introduced in which they are much less sensitive to tilt and more sensitive in detecting local curvature changes^34^. The tangential map is defined by the curvature of the corneal meridian at a point on a surface. Here, tangential maps were developed for clinical validation procedures.

For the reconstruction of the topographic maps, corneal elevation data was exported in a comma-separated file format (CSV) from Pentacam and then was imported into Matlab for processing. The Matlab code provides point cloud data for each corneal surface in the form of $Z_{i}=Z\left( x_{i}, y_{i}\right)$ where $Z_{i}$ is the corneal height data in mm on a 0.1 mm grid at $\left( x_{i}, y_{i}\right)$ spacing. Then, 8th Zernike polynomials were fitted to reconstruct the anterior corneal surface from the obtained point clouds:

4 $$\begin{aligned} Z_{n}^{m}(\rho , \varphi )=R_{n}^{m}(\rho )\left\{ \begin{array}{ll} \cos (m \varphi ) &{} \text{ if } m \text{ is } \text{ positive } \\ \sin (m \varphi ) &{} \text{ if } m \text{ is } \text{ negative } \end{array}\right. \end{aligned}$$

where $\rho =\left( x^{2}+y^{2}\right) ^{1 / 2}$ is the normalized radial distance (from a surface point on the projected $x y$ plane to the optical axis, which was assumed to be aligned with the axis of vision). In this equation, $0<\rho \le 1$ and $\varphi$ is the azimuthal angle. The non-negative integer $n$ is the radial order. The integer $m$ indicates the azimuthal order, where $n-m$ must be even. The radial polynomials $R_{n}^{m}$ are defined by:

5 $$\begin{aligned} \begin{aligned} R_{n}^{m}(\rho )=\sum _{k=0}^{\frac{n-m}{2}} \frac{(-1)^{k}(n-k) !}{k !\left( \frac{n+m}{2}-k\right) !\left( \frac{n-m}{2}-k\right) !} \rho ^{n-2 k} \end{aligned} \end{aligned}$$

Once the Zernike polynomials for the anterior corneal surface were computed, the principal curvatures of the cornea can be estimated. The analytical expressions of axial and tangential curvatures using the Zernike polynomials of the first and second derivatives were derived by^35^:

6 $$\begin{aligned} \begin{aligned} \kappa _{\text {sag}}=\frac{d Z / d \rho }{\rho \left[ 1+(d Z / d \rho )^{2}\right] ^{1 / 2}} \end{aligned} \end{aligned}$$

7 $$\begin{aligned} \begin{aligned} \kappa _{\text {mer}}=\frac{d^{2} Z / d \rho ^{2}}{\left[ 1+(d Z / d \rho )^{2}\right] ^{3 / 2}} \end{aligned} \end{aligned}$$

**Figure 8:**
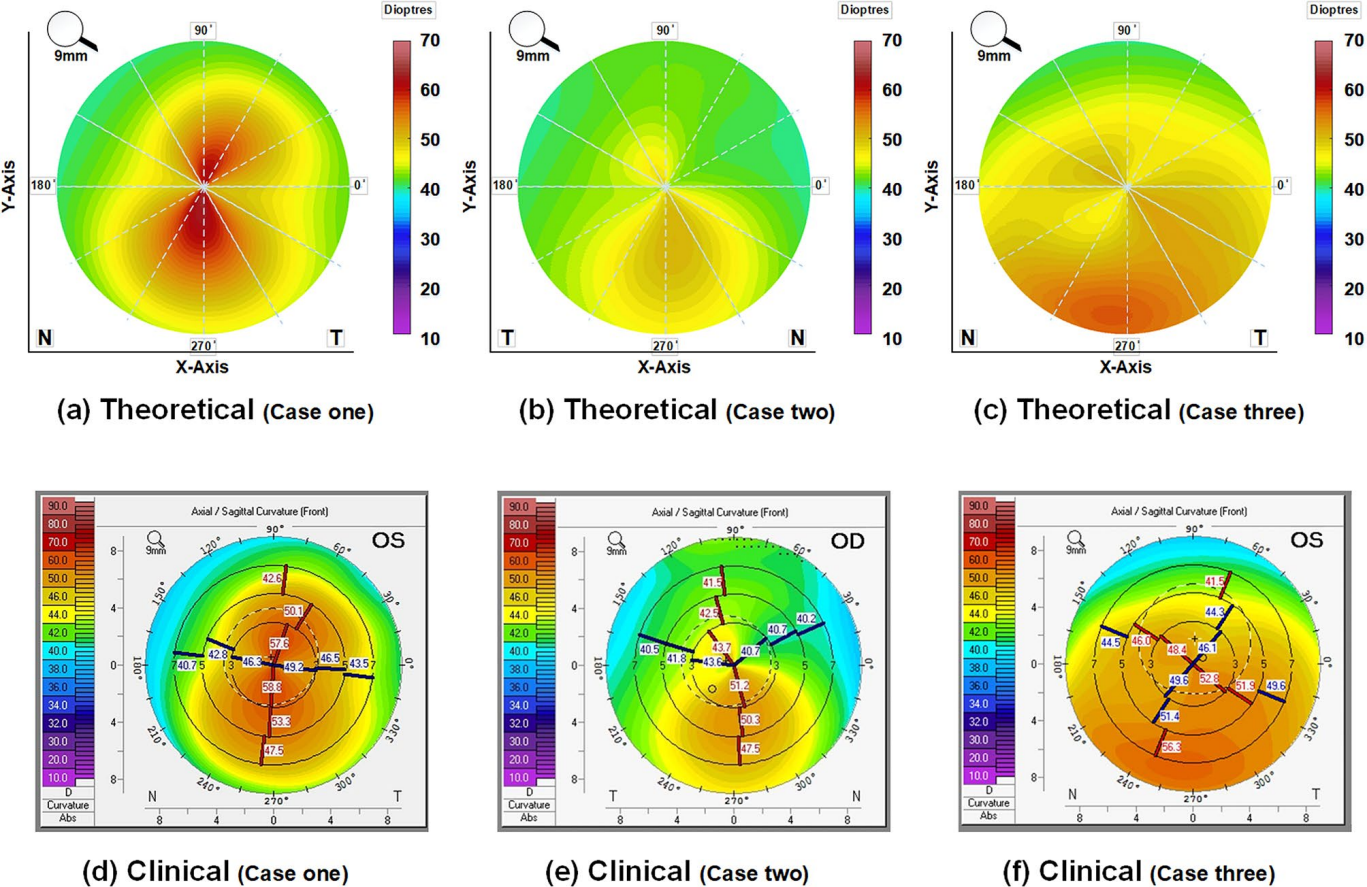
The preoperative axial maps: theoretically obtained from Pentacam elevation data for three individual case scenarios (the first row); the clinically reported axial maps for each case (the second row).

The expressed curvature values were converted into diopters (D) using Snell’s law and a corneal refractive index of 1.3375^36^. Figure 8 (first row) presented the preoperative axial maps of three different keratoconus patterns, including symmetric bow-tie (case one), oval (case two), and pellucid-like (case three), which were theoretically derived from the evaluation data of the Pentacam data of each patient. These were in good agreement with Fig. 8 (second row), which indicated the clinically reported preoperative axis maps of the patient.

### The 3D patient-specific FEM of keratoconus cornea

**Figure 9:**
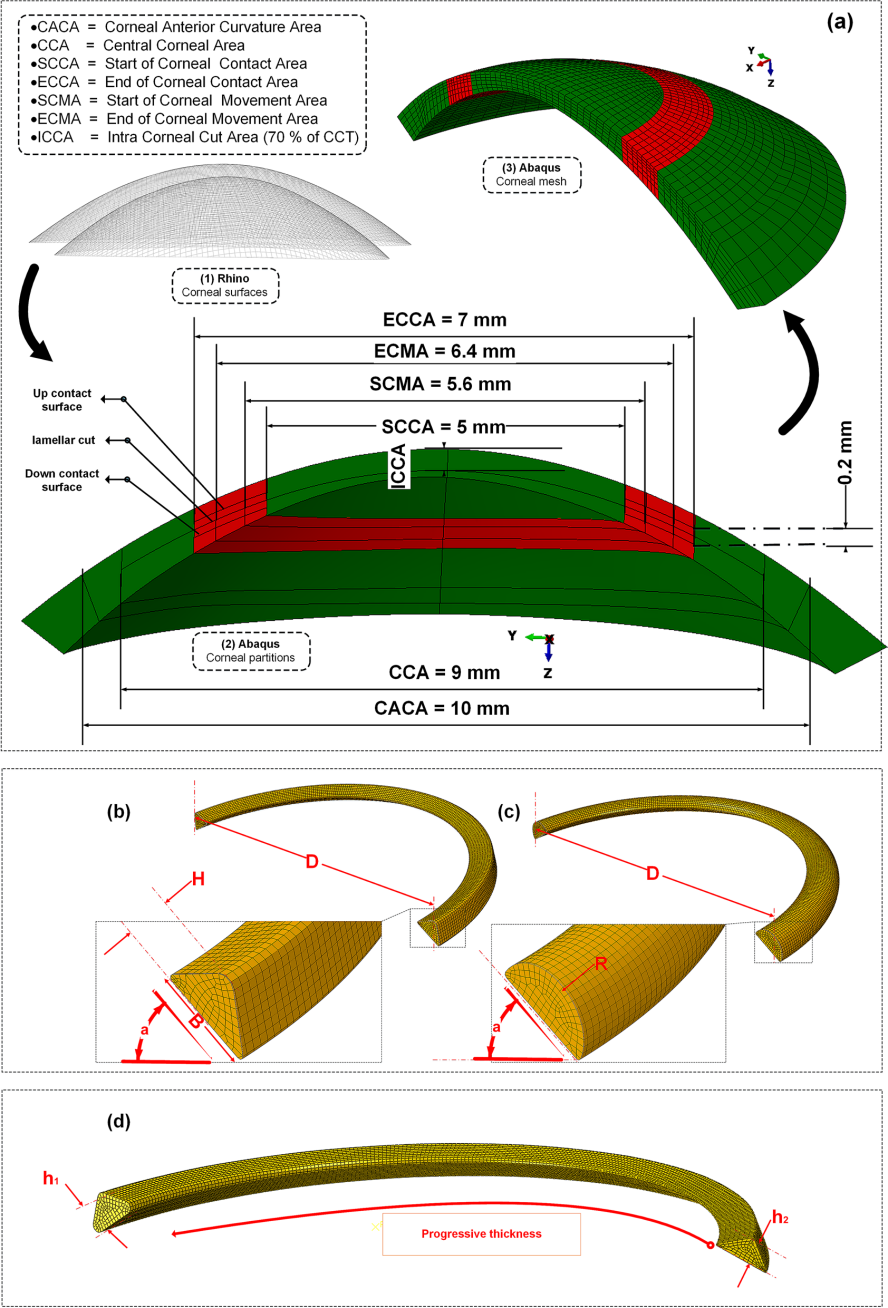
(**a**) The patient-specific 3D construction of the cornea FEM. The defined variables of developed ICRS in 3D FEM (**b**) KeraRing, (**c**) MyoRing, (**d**) progressive KeraRing.

In a first step, the patient’s corneal height data obtained by Pentacam tomography (Oculus Inc., Wetzlar, Germany) were imported into Matlab to reconstruct point clouds of corneal surfaces. The derived point cloud surfaces were then exported to the surface reconstruction software RhinocerosÒ V 5.0 (McNeel & Associates, Seattle, USA) (Fig. 9a(1)). This software uses a mathematical model to generate surfaces using non-uniform Rational B-Splines (NURBS). In an eralier research, its efficiency and accuracy in corneal surface reconstruction is evaluated^37^. The corneal geometric parameters derived from each patient clinical data, were listed in Table 4. The software ABAQUS® (version 6.17, Dassault Systemes, France) was used to create a 3D model of the cornea and the ICRS. By assigning anisotropic hyperelastic material model and corneal intracorneal pressure (IOP), a Python iteration based function was employed to obtain the stress-free configuration of the cornea^38^.

**Table 4:** Characteristics of the cornea in different cases.

Case	Pachy. min (mm)	Front Rh (mm)	Front Rv (mm)	Max. diameter (mm)
No.1	389	7.06	5.84	11.96
No.2	497	7.94	7.17	12.31
No.3	424	7.06	6.68	12.6

As explained, the 3D corneal model was created using computer-aided design software (Rhinoceros) and FEM software (Abaqus), as shown in Fig. 9a(2). It was assumed that an incompressible, anisotropic, nonlinear, hyperelastic material model represents the keratoconus material model. Modified Gasser-Holzapfel-Ogden (GOH), with the strain energy function from the original GOH model^39^ with pre-integrated fiber distribution, is used. The modified strain energy function per unit of a reference volume, $U$, is defined as follows:

8 $$\begin{aligned} \begin{aligned} U & =C_{10}\left( {\bar{I}}_{1}-3\right) +\frac{1}{D}\left( \frac{J_{e l}^{2}-1}{2}-\ln \left( J_{e l}\right) \right) \\ & \quad +\frac{k_{1}}{2 k_{2}} \sum _{\alpha =1}^{N}\left\{ \exp \left[ k_{2}\left\langle {\bar{E}}_{\alpha }\right\rangle ^{2}\right] -1\right\} \end{aligned} \end{aligned}$$

where $C_{10}$ and $k_{1}$ are stress-like parameters relating to matrix stiffness and fiber stiffness, respectively; $k_{2}$ is a dimensionless parameter relating to fiber nonlinearity. ${\bar{I}}_{1}$ is the first invariant of the modified right Cauchy-green tensor. $J^{e l}$ is the elastic volume ratio and $D$ is an incompressibility penalty constant, accordingly the corneal incompressibility, which was set to $1 \times 10^{-3} \; \text {MPa}^{-1}$;

9 $$\begin{aligned} \begin{aligned} {\bar{E}}_{\alpha } {\mathop {=}\limits ^{ \text{ def}}} \kappa \left( {\bar{I}}_{1}-3\right) +(1-3 \kappa )\left( {\bar{I}}_{4(\alpha \alpha )}-1\right) \end{aligned} \end{aligned}$$

The parameter $\kappa (0 \le \kappa \le 1 / 3)$ expresses the degree of dispersion in the fiber directions. $\rho (\theta )$ is the orientation density function, which characterizes the distribution of the fibers in the reference configuration $\Omega _{0}$ with respect to the mean orientation in the range of $[\theta , \theta +d \theta ]$. Thus $\kappa ,$ is defined as follows:

10 $$\begin{aligned} \begin{aligned} \kappa =\frac{1}{4} \int _{0}^{\pi } \rho (\theta ) \sin ^{3} \theta d \theta \end{aligned} \end{aligned}$$

In another word, the parameter $\kappa$ describes the fiber dispersion with respect to the mean fiber orientation at any given location; $\kappa =0$ for perfect alignment, and $\kappa =\frac{1}{3}$ for random distribution, and the material becomes isotropic. In this study, $\kappa$ was set to zero for perfect alignment. In a recent study by^14^, the patient-specific material behavior of the keratoconic and healthy cornea was evaluated using an extensive database. The study showed that while differences in the isotopic terms of the strain energy had meaningful effects on the simulated corneal displacement, anisotropic terms including k1, and k2 did not. Therefore, in the present study $k_{1}$ and $k_{2}$ were set to the average values of 0.019 MPa and 250. $C_{10}$ were selected in three specific descending quantities to evaluate the contribution of these constants to keratometry and the induced biomechanical state. The constant material properties of the cornea of each patient were presented in Table 5.

**Table 5:** Holzapfel material constants in different cases.

Case	C_10_ (MPa)	k_1_ (MPa)	k_2_
No.1	0.4	0.019	250
No.2	0.3	0.019	250
No.3	0.2	0.019	250

As shown in Fig. 9a(3), the corneal mesh was created with linear hexahedral elements of the C3D8R type. The method of iterative net sensitivity analysis, which contains more than 25,000 elements, was also considered. Limbus, iris, and sclera were excluded since no precise information about their mechanical properties were available and the inclusion of additional parts brought more uncertainties into the simulation results^40^. Therefore, the displacement of the corneal nodes in the limbus was constrained, as a boundary condition.

### The 3D FEM of various ICRS design

Two types of widely used polymethylmethacrylate (PMMA) ICRS were used in FEM based treatment planning, known as KeraRing and MyoRing implants. The 3D model of each type of ICRS with the anatomy and dimensions corresponding to the reported clinical cases were listed in Table 6. The angle of the two ICRS bases with a horizontal axis was set at 30 degrees. The assumed ICRS variables were listed in Table 6, and there are shown in Fig. 9b–d. To present a comparative study, the KeraRing dimensions were selected similarly to the corresponding MyoRing characteristics in the respective scenarios.

**Table 6:** Variables of ICRS in different cases.

Case	B (mm)	H (mm)	R (mm)	D (mm)	h1 (mm)	h2 (mm)
No.1	0.43	0.28	0.28	6	0.28	0.18
No.2	0.3	0.25	0.25	6	0.25	0.15
No.3	0.43	0.3	0.3	6.2	0.3	0.2

The ICRS was modeled as linear elastic and isotropic, with a Poisson’s ratio of 0.3 and a modulus of elasticity of 3100 MPa. The linear hexahedral elements of type C3D8R were generated in those FEM with more than 15,000 elements.

### FEM assumptions according to the clinical analysis

**Figure 10:**
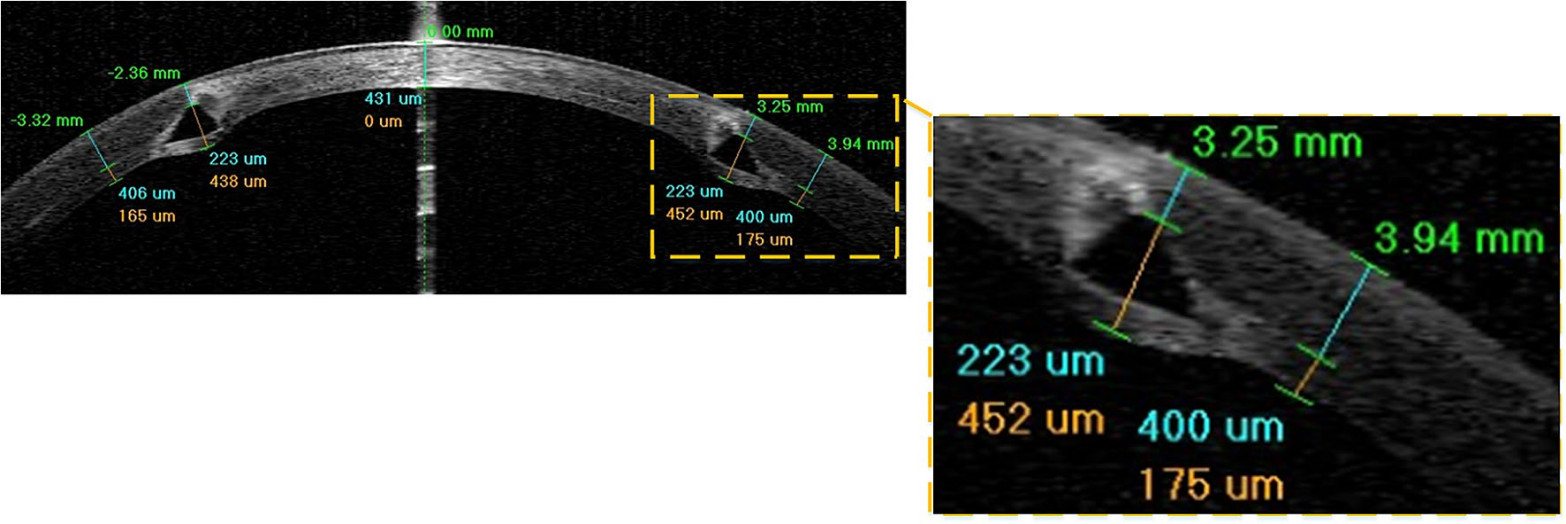
The postoperative AS-OCT image of ICRS implementation surgery (case three).

To interpret the definitions of ICRS implantation surgery in the FEM, postoperative AS-OCT images at different stages of keratoconus were carefully studied. The postoperative AS-OCT images show that although the ICRS are implanted with the upper and lower surface facing the corneal layer, the ICRS have penetrated the corneal stroma layer in the contact area, as shown in Fig. 10. Occasionally, a white deposit of fatty acids was reported at the sites of implant-corneal interaction, which does not cause visual changes^41^. One of the major challenges was to introduce this penetration and interaction complexity into the FEM definitions. However, no information was available on the value of these regional penetrations nor the characteristics of the material failure in the deposits. To overcome this problem, a simplification was introduced instead of dealing with an unknown damage and failure model, which could lead to additional uncertainty. As shown in Fig. 9a(2), two contact surfaces were specified at a distance of 0.1 mm from the pocket surface in the upper and lower direction according to the implant penetrations. Therefore, the interaction of the ICRS with these surfaces was similarly defined in all case scenarios.

### Patient-specific ICRS implementation surgery simulation

**Figure 11:**
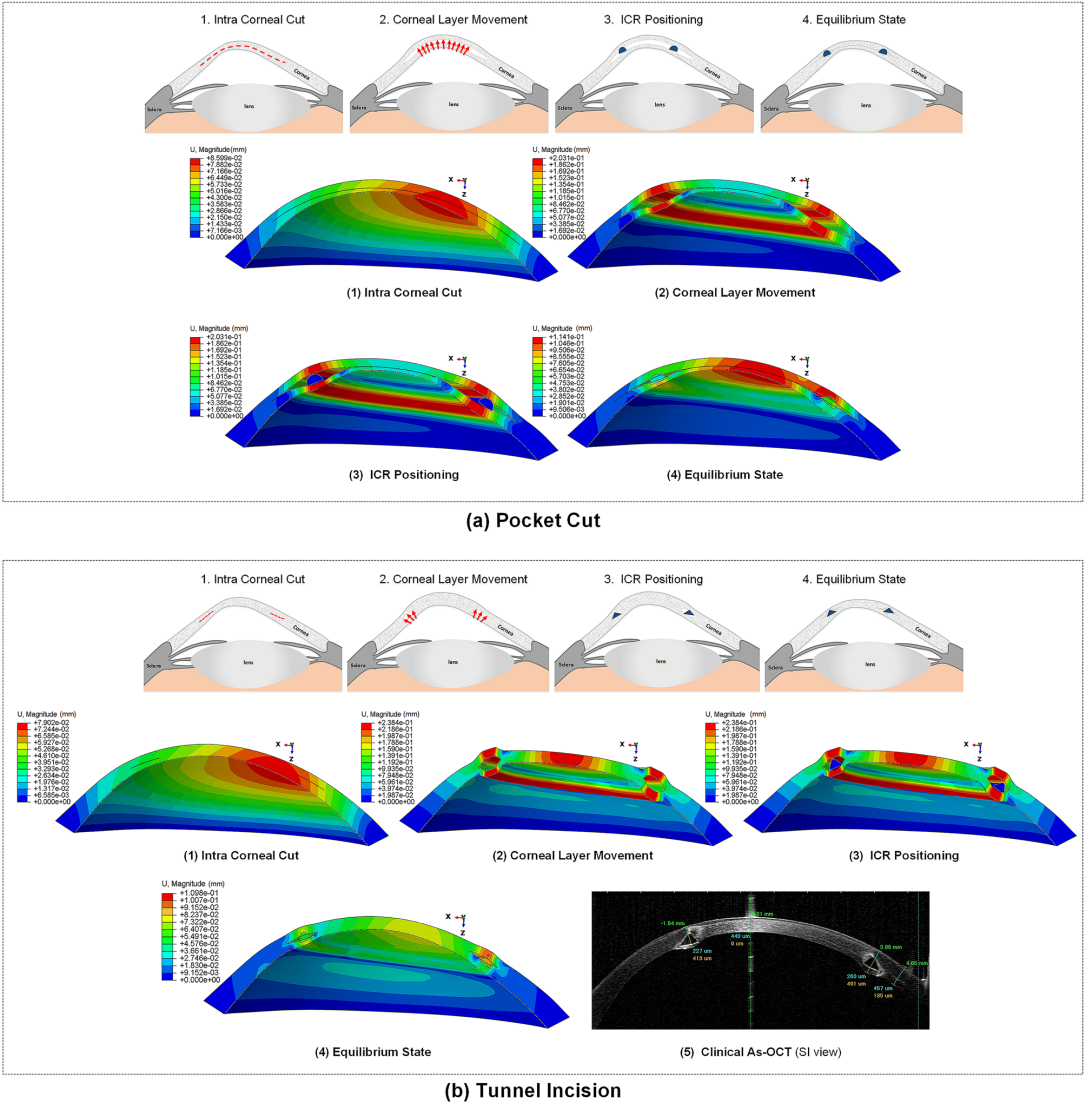
The summary of the step-by-step ICRS implementation methods; (**a**) pocket cut; (**b**) tunnel incision.

According to the ICRS surgical procedure, there are two main implementation methods prescribed by the insertion of a tunnel incision and a lamellar pocket cut. In the present study, to define the operation in finite-element steps, the surgery was divided into four general phases. The schematic sequence of the lamellar pocket cut steps shown in Fig. 11a were detailed as follows:

Step 1.: The PocketMaker ultraKeratome or femtosecond laser was used to create a lamellar cut in the corneal stroma, which represents a virtual gap of 9 mm diameter and 300 μm depth.
Step 2.: In a 4 mm wide temporal lamella site, the upper layer of the corneal layer was moved with an open up forceps prior to the ICRS insertion.
Step 3.: The ICRS were positioned centrally within the corneal pocket according to the manufacturer’s guidelines, using the intersection of the optical axis and the corneal surface as a reference.
Step 4.: Since no suture was required, the ICRS and cornea were left to create their new equilibrium.

At the same time, the steps of the ICRS operations with a tunnel incision, as shown in Fig. 11b, were summarized as follows:

Step 1.: A femtosecond laser was used to make a tunnel incision with a preset inner and outer diameter at 300 μm corneal depth.
Step 2.: The opening of the incision access with a Sinskey Hook up to the height of the ICRS was presented.
Step 3.: ICRS were introduced into the tunnel by a simultaneous thrust with a McPhersonian forceps.
Step 4.: The cornea and ICRS were stabilized to new configurations.

The first step in the development of FEM of surgery is to apply hydrostatic pressure to the cornea caused by fluids inside the eye. The same reference value of physiological intraocular pressure (16 mmHg) was assumed for all three cases. Subsequently, the corneal pocket cut with a diameter of 9 mm and the tunnel incision with an inner diameter of 5 mm and an outer diameter of 7 mm, both at 300 μm corneal depth, were created by a “seam” option in the interaction module of Abaqus/CEA. According to the intracorneal microstructure, the contact behavior between the intracorneal layer was defined as “frictionless” and “hard” contact. The pressure structure of the cornea with induced lamella pocket and tunnel incision was shown in Fig. 11a(1),b(1). According to the clinical instructions, the intra-layer of the cornea moved by about half of the ICRS height to introduce a gap for ICRS implementation. The movement of the layers under physiological IOP was defined by displacement boundary conditions for the upper and lower corneal layer (Fig. 11a(2),b(2)).

**Figure 12:**
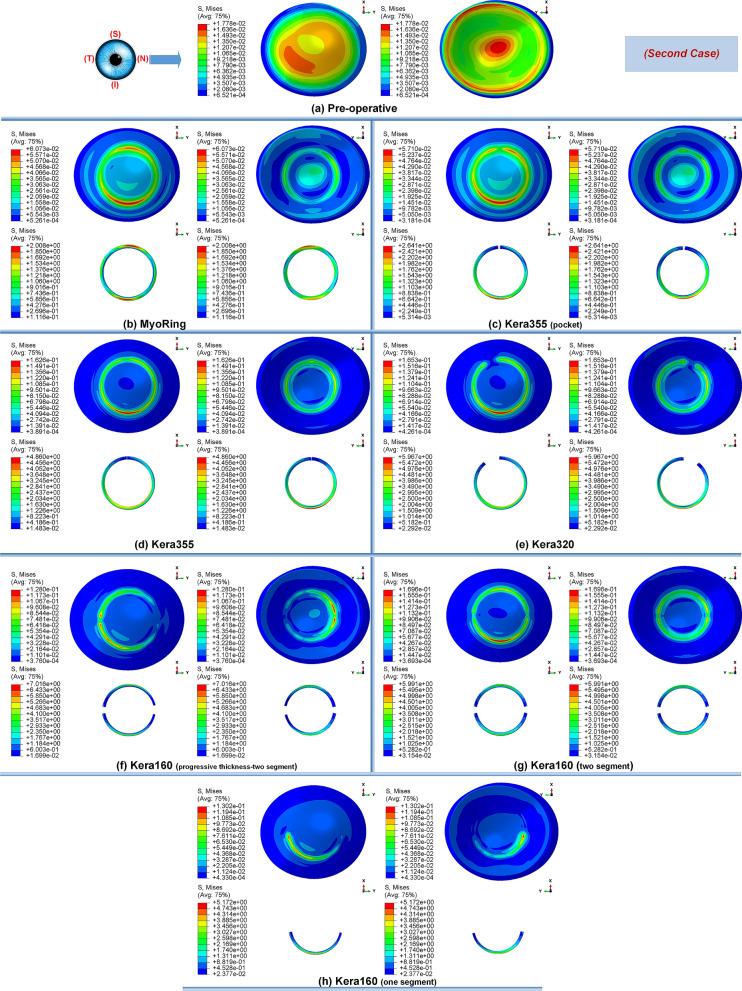
The pre- and post-operative contour diagrams of the Von Mises stress distribution (MPa) in FEM of different surgical scenarios (second case): (**a**) preoperative; (**b**) MyoRing; (**c**) Kera355 implemented by pocket incision; (**d**) Kera355; (**e**) Kera320; (**f**) Kera160-two prog segments; (**g**) Kera160-two segments; (**h**) Kera160-one prog segment.

**Figure 13:**
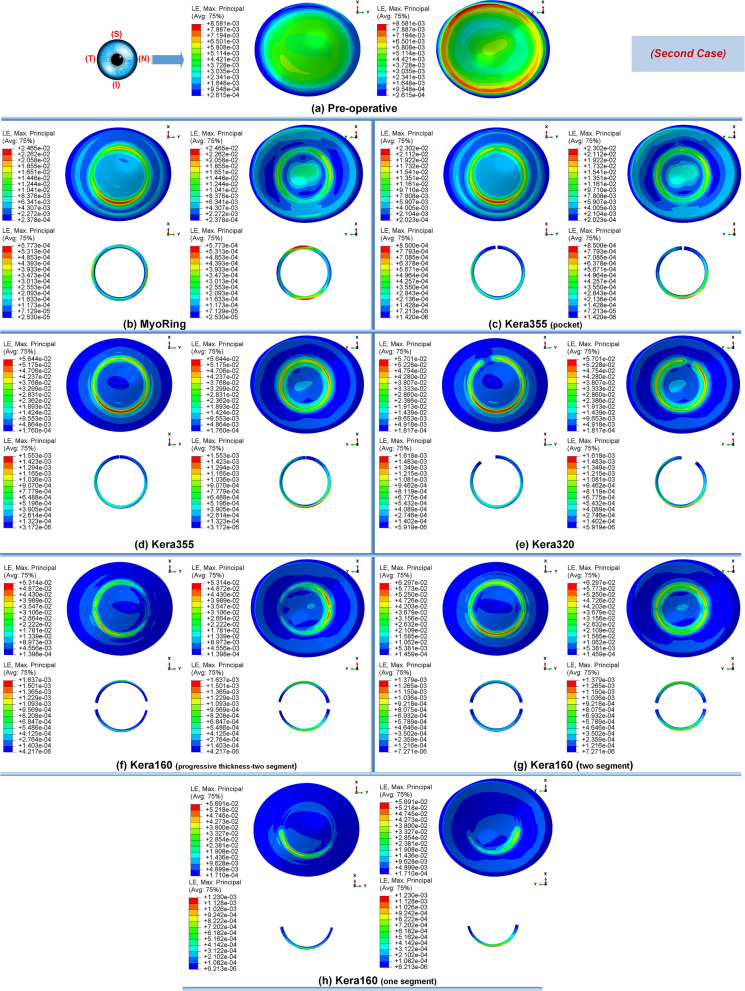
The pre- and postoperative contour plots of the principal strain’s distribution in FEM of the different surgical scenarios (second case): (**a**) Preoperative; (**b**) MyoRing; (**c**) Kera355 implemented by pocket incision; (**d**) Kera355; (**e**) Kera320; (**f**) Kera160-two prog segments; (**g**) Kera160-two segments; (**h**) Kera160-one prog segment.

**Figure 14:**
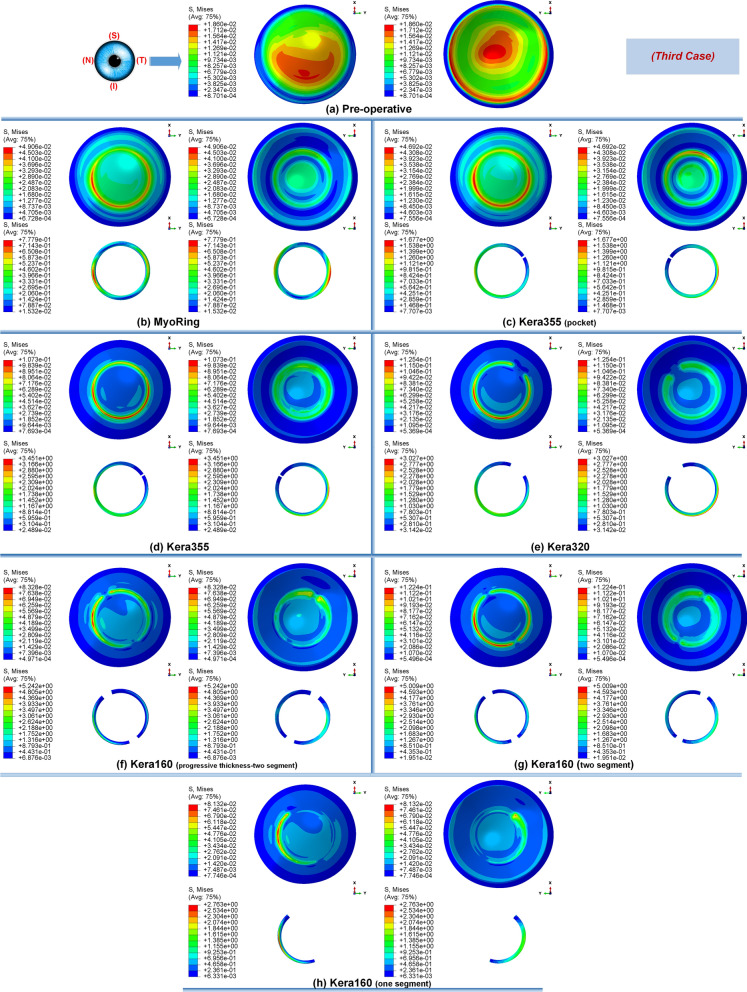
The pre- and post-operative contour diagrams of the Von Mises stress distribution (MPa) in FEM of different surgical scenarios (third case): (**a**) preoperative; (**b**) MyoRing; (**c**) Kera355 implemented by pocket incision; (**d**) Kera355; (**e**) Kera320; (**f**) Kera160-two prog segments; (**g**) Kera160-two segments; (**h**) Kera160-one prog segment.

**Figure 15:**
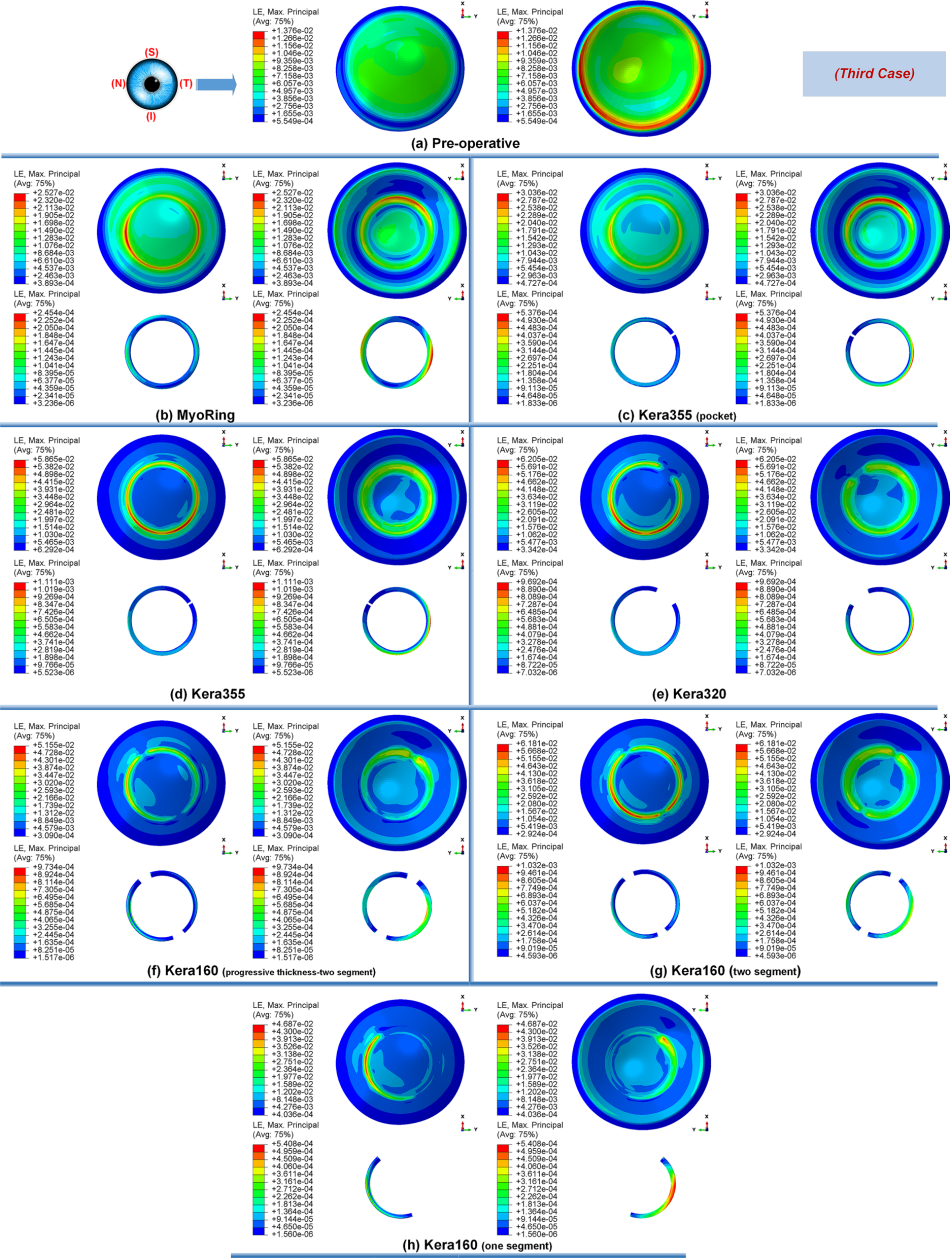
The pre- and postoperative contour plots of the principal strain’s distribution in FEM of the different surgical scenarios (third case): (**a**) Preoperative; (**b**) MyoRing; (**c**) Kera355 implemented by pocket incision; (**d**) Kera355; (**e**) Kera320; (**f**) Kera160-two prog segments; (**g**) Kera160-two segments; (**h**) Kera160-one prog segment.

The ICRS were positioned and centered within the corneal induced gap (corresponding to corneal tilt and misalignments) according to the AS-OCT data of the individual patient. In the third step of the analysis, a surface-to-surface contact interaction, defined as finite-sliding, and the contact properties “frictionless” and “hard” were defined for the implant-cornea interactions. To simulate the effect of implant repositioning, which leads to the generation of compressive and tensile stresses on the implant, the ICRS was not restricted and left to find their stabilized configuration. At the same time, the displacement boundary conditions on the moving corneal layers were gradually removed to introduce implant-interlamellar contact interaction (Fig. 11a(3),b(3)). All steps were designed in Abaqus/Standard, a robust implicit solver, to perform a highly precise static process. In the final step of the analysis, all displacement limits were removed, leaving an unconstrained implant-corneal interaction to restore the final balance (Fig. 11a(4),b(4)). To provide a clinical perception of the developed FEM, the post-operated AS-OCT image of the evaluated cornea is reported in Fig. 11b(5). To examine the effects of implant design, five different types of KeraRing were simulated and compared in all three clinical cases using tunnel cut methods to replicate their deployment configurations. The finite-element procedure of ICRS implementation surgery for both methods (tunnel incision and lamellar pocket cut) along with the further post-operated corneal stress and strain analysis of different cases are shown in Figs. 12, 13, 14 and 15.

## Supplementary Information

Supplementary Video S1.

Supplementary Video S2.
